# Supplementary figures and images for: Genome-wide association analysis uncovers the genetic architecture of tradeoff between flowering date and yield components in sesame
Source: BMC Plant Biol. 2021 Nov 22;21:549. doi: 10.1186/s12870-021-03328-4 (PMC8607594; doi:10.1186/s12870-021-03328-4)

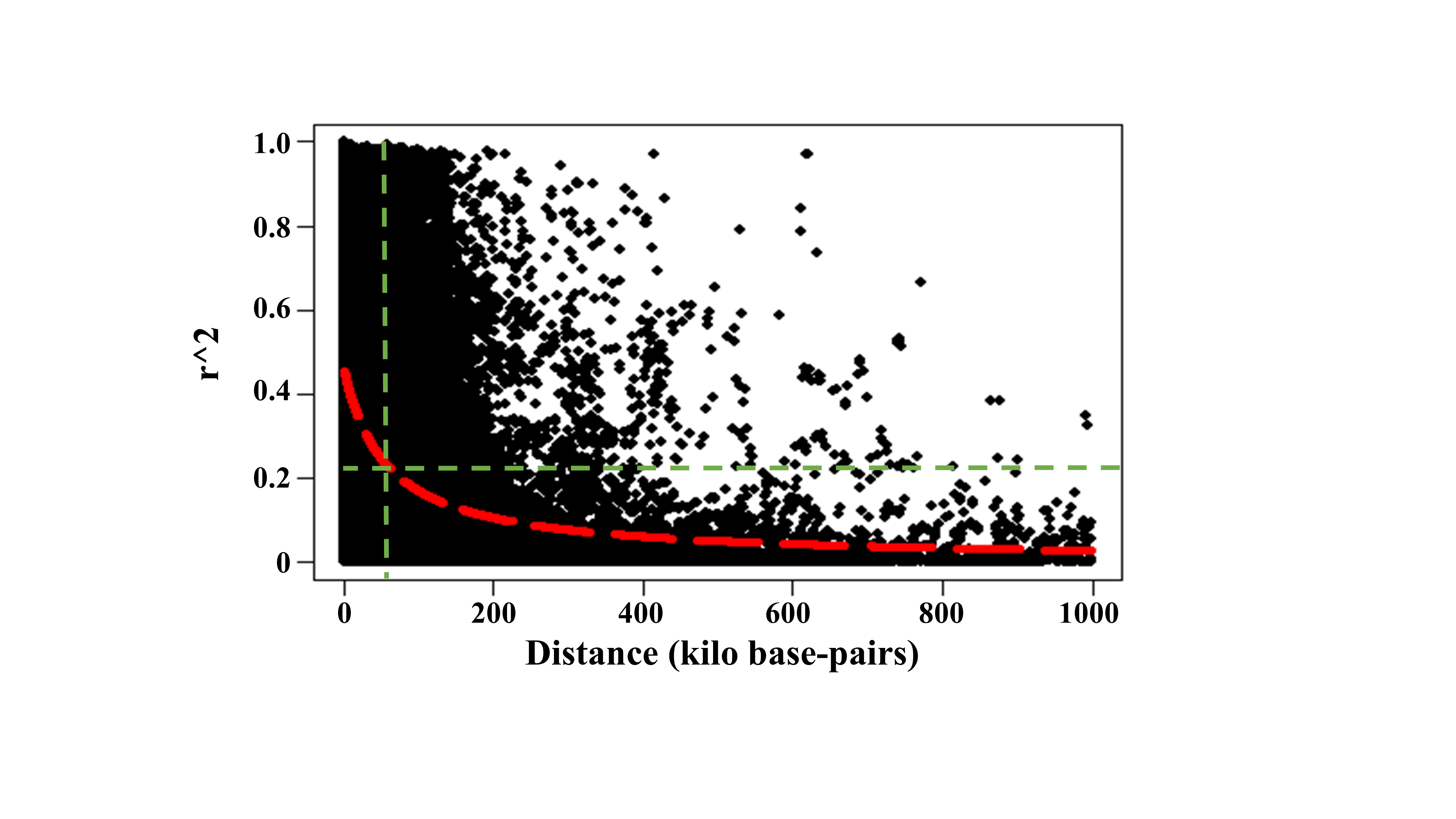

Supplement: Supplementary file 3 — Additional file 3: Fig S2. Genome-wide linkage disequilibrium pattern in the SCHUJI panel. The red dashed line represents the non-linear trend and the green dashed line indicates the point where linkage disequilibrium drops to half of its initial value (0.22) at 58,774 base pairs. [file 12870_2021_3328_MOESM3_ESM.tif]

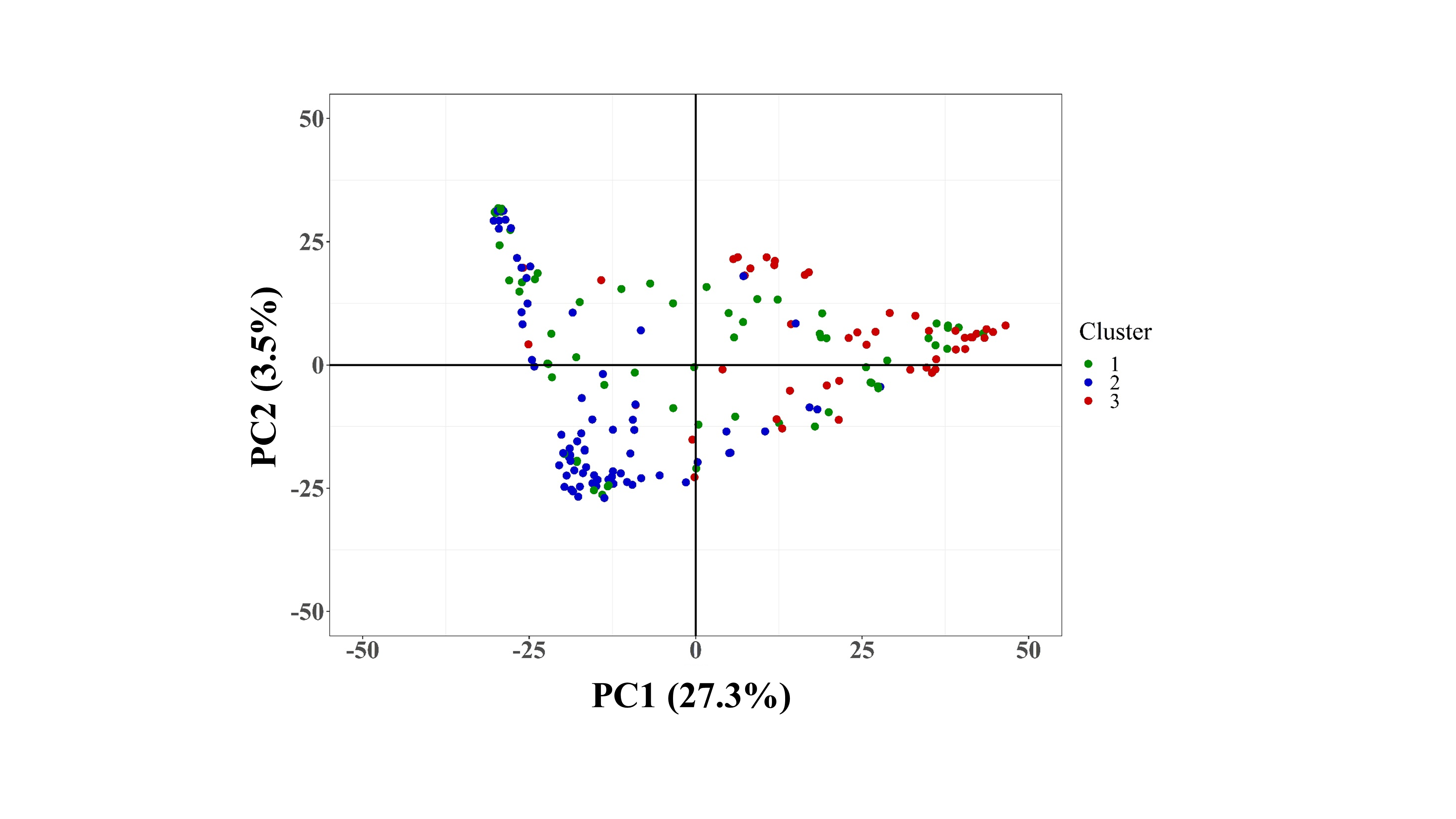

Supplement: Supplementary file 5 — Additional file 5: Fig S4. Principal component analysis on genetic markers of the SCHUJI panel. Colors represent different clusters from the k-means cluster analysis. Red color represents cluster 3, green color represents cluster 1 and blue color represents cluster 2. [file 12870_2021_3328_MOESM5_ESM.tif]
